# Supplementary material for: Sex differences in the fecal microbiome and hippocampal glial morphology following diet and antibiotic treatment
Source: PLoS One. 2022 Apr 6;17(4):e0265850. doi: 10.1371/journal.pone.0265850 (PMC8985946; doi:10.1371/journal.pone.0265850)
Supplement: Attachment — Submitted filename: Response to Reviewers.docx [file pone.0265850.s001.docx]

**Response to Reviewers:**

**We would like to thank the reviewers for their time, comments such as “this was an interesting paper and is well written”, and their thorough review in order to improve our manuscript. We have made substantial revisions throughout the paper, as outlined below and found in our track changes document.**

**Reviewer 1:**

One of the main problems of the manuscript is that – even though the authors state several times the the gut-brain-axis might play a crucial role (Abstract, line 21, and line 78 in Intro) – it was not examined.

**We have removed this from the manuscript and have now listed this as a limitation for our study as well as a goal for future studies.**

• The Abstract is very basic, it does not say which animals were used. Which antibiotics, for how long…..

**We have now added specific information about the animals and treatments to the Abstract.**

• Line 42: The CDC reference should be added as a reference.

**We have added this reference.**

• Line 59: Please describe here whether the relative abundance of Firmicutes and Bacteroidetes increases or decreases due to high fat diet.

**We have clarified that Firmicutes is increased and Bacteroidetes is decreased, supported by our citations.**

• Line 83: The authors describe the use of antibiotics as an aim without writing a word about them in the Introduction.

**We have added a paragraph about the use of antibiotics as well as clarified their use in this study by stating our hypothesis.**

• The last two sentences of the introduction (line 84-87) should be deleted. They do not belong to the Introduction.

**Thank you. This has been deleted.**

• Line 106: I think that “..to a subgroup of mice” is confusing. Does this form a new group? Or is this just the antibiotic group. If the second one is true, then the statement could be removed.

**Thank you. This has been deleted.**

• Line 108: How was food consumption measured? Metabolic cages or weighing the food?

**We have clarified in the text that food consumption was determined by manually weighing the food.**

• Line 116: How were the fecal samples stored until analysis?

• Line 116: It would be helpful to add the information when the last day of study was. Same for line 142.

**For the two points above, we have added this to the text (underlined portion): Fecal samples were collected using sterile technique on the last day of the study (after 16 weeks of diet administration, at the time of euthanization) and stored in microcentrifuge tubes at -80^o^ C until analysis.**

• Statistical Analysis, line 186 and 187: The authors have used an ANOVA. Were the data normally distributed? With the relatively small number of animals per group a non-parametric comparison would be more appropriate.

**Thank you for drawing our attention to this. We have now conducted a Shapiro-Wilk test for these data. The body weight data were not normally distributed. We performed a log transformation of the data and these data were normally distributed. We re-analyzed the log transformed data with a two-way ANOVA and have adjusted the F and p-values, accordingly. This has been updated in the Results section and clarified in the Methods section.**

• Results, lines 196-198: These are not really results and should be shown in the Methods section.

**Thank you. We moved this to the Methods section.**

• Results, line 201: Do you mean the caloric consumption over the whole study period?

**Yes. We have added “over the whole study period” to this sentence for clarity.**

• Results, lines 202 and 203: Are the values shown somewhere? Do the authors mean the summarized calories over the whole study? This is unclear to me.

**We recognize that this added data may cause confusion. We performed a three-way ANOVA for sex x treatment x time for caloric consumption as well as a two-way ANOVA for average food consumption, for the entire study period. We have removed a few sentences in order to improve clarity and only deliver the essential information.**

• Results, lines 203-205: This is not a results but a Discussion.

**Thank you. We have moved this to the Discussion.**

• Results, lines 205-210 and Figure Caption 1A: This is a lot of redundant enumeration. The authors should consider to add some marks the Figure 1A depicting the statistically significant differences. Same for Figure 1B.

**We have edited the caption as well as Figures 1A and 1B.**

• Figure Caption 1C: This is not weight gain but weight. Please correct.

**Thank you for drawing our attention to this error. We have corrected it.**

• Figure 1: Are the values shown mean and SD or medians and IQR. How were the data distributed?

**We have added that the data reported are mean +/- SEM. We have now clarified which data are normally distributed and any corrections in the Methods section.**

• Figure 1A and B: Either use “LF” or “low fat”. And “HF” or “high fat”

**We have corrected this error.**

• Figure 1C: What I do not understand is that at week 0 the male HF antibiotics group had about 25 grams mean compared to female LF animals with 17 grams? Please clarify. The weight at week 0 should be more or less the same for all groups. This is confusing.

**This is typical for males vs. females at 2 months of age, in our previous studies as well as studies from other laboratories. Female mice tend to be smaller than male mice** [**https://www.jax.org/jax-mice-and-services/strain-data-sheet-pages/body-weight-chart-000664#**](https://www.jax.org/jax-mice-and-services/strain-data-sheet-pages/body-weight-chart-000664)

**We included results for weight gain to show change from week 0 to week 16 (Figure 1B) as well as weekly weights in order to show the trajectory for each group (Figure 1C).**

• Microbiome analysis: I would prefer to read to alpha-diversity first, followed by beta-diversity and relative abundances.

**We agree with your point and have edited the text (and Figures) to present the data in that order.**

• Table 1: I think Table 1 is confusing and would prefer a Figure showing the relative abundances.

**We have removed Table 1 and have now included a Figure for the top 14 most abundant genera of bacteria.**

• Lines 240-242: Please refer to the respective Figure here. Do the results remain statistically significant (female LF antibiotics vs. all other antibiotic groups) when comparing all 8 groups?

**We have edited the text to refer to Figure 2A here. Yes – this statistical analysis was performed including all 8 groups, and revealed statistically significant differences for female LF antibiotics compared to all other antibiotic groups.**

• Many of the changes found in relative abdundances are below 3%. The biological relevance can be questioned and should be discussed.

**As done by Nagpal et al. (**[**https://www.mdpi.com/1422-0067/21/10/3434/htm#B33-ijms-21-03434**](https://www.mdpi.com/1422-0067/21/10/3434/htm#B33-ijms-21-03434)**), we have now excluded data below 0.5% mean relative abundance. The 0.5% abundance may only be met by one of the eight groups. For any genus that did not meet the 0.5% criteria by any group, we have removed it from the table and analysis. Also, we have now included Figure 5 to display the top 14 most abundant genus of bacteria visually. However, we agree that a next step to this, and in future analyses, the biological relevance of the particular genus and threshold abundance for specific impact on metabolite production, etc. will be important. We aim for these data to inform future studies.**

• Line 276: delete “as we expected”.

**This has been deleted.**

• Caption Figure 3: This is alpha-diversity and should be added.

**‘Alpha diversity’ has been added.**

• Lines 286-294: These are rather Methods and should be described there.

**Thank you. We moved this to the Methods section.**

• Figure 5: There is no A etc. shown in the Figure.

**We have edited the Figure to include A-E.**

• The description of Figure 5 in the text and in the caption is redundant and could be shortened. Moreover, in the caption there is no explanation of “*” etc.

**We edited the caption to remove redundancy and have included explanation for the asterisks.**

• Line 335: How do we see the distinct clustering of the two groups, there are all dots?

**We have edited this caption and now refer to “results with low Shannon diversity for antibiotic treated vs. higher Shannon diversity for mice that received normal drinking water”**

• The Discussion does not follow the line of the results. This should be changed.

**We agree with this point and have edited the text and flow of the Discussion.**

• There is no conclusion section. Moreover, the authors should include a limitations of the study section.

**We have added a paragraph about limitations and a Conclusion section.**

• The authors have examined the fecal microbiome and not the gut microbiome. This should be changed throughout the manuscript.

**Thank you for drawing our attention to this error. We have updated our reference to our analyses from “gut microbiome” to “fecal microbiome”. We do still refer to ‘gut microbiome’ generally in the Introduction and Discussion, but whenever we refer to our analyses and results, we state ‘fecal microbiome’.**

Reviewer #2: PONE-D-21-28243
Abstract/Introduction:
1. The abstract needs to summarize the findings and implications of the study better.
**We have edited the Abstract to include more details for Methods and Results.**

2. Overall, the introduction is well written. However, the rationale for sex differences by summarizing past research was underdeveloped and could use a stronger summary.

3. The specifics from prior research summarizing the links between the key variables was missing.
4. The hypotheses were not specific. What do the authors expect to observe based on the prior literature?
**Thank you. For points 2-4, we have edited the Introduction. We now include more information about the use of antibiotics, established sex differences, and a specific hypothesis.**


Discussion and Conclusion
1. However, it is still not clear the reason behind the opposite effects observed in males and females as a result of diet and antibiotics and hippocampus glial morphology. More discussion of these results needs to take place. These results seem counterintuitive to past research and to the diet*sex results the authors found.
2. There are a few overstatements of the results. Most of the discussion centers around summary of the results.
3. Most importantly, what do these findings mean? How is this important? Discussion around the integrated results is needed to make these findings meaningful.

**For points 1-3, we have made significant changes to the Discussion section. We now discuss previous studies and how our results agree/contradict with those studies. We also now include discussion on what the difference in microglial morphology could be caused by (metabolic factors). Please see our track changes document for all of these changes.**

4. Minor: the images were slightly blurry. The Double Dendrogram image was extremely difficult to read.

**We have uploaded new images; they meet the suggested resolution as indicated by the journal so we hope that they no longer appear blurry.**
