# Supplementary material for: Sex differences in the fecal microbiome and hippocampal glial morphology following diet and antibiotic treatment
Source: PLoS One. 2022 Apr 6;17(4):e0265850. doi: 10.1371/journal.pone.0265850 (PMC8985946; doi:10.1371/journal.pone.0265850)
Supplement: Attachment — Submitted filename: Response to Reviewers-revision 2.docx [file pone.0265850.s002.docx]

**Response to Reviewers:**

**Thank you for your time and thorough review. Please find our response to the specific comments, below. We have also included a track changes document.**

Reviewer 1: The authors have answered the questions and concerns and the manuscript has significantly improved. However, a couple issues remain:

1) Abstract: The study aims should be presented prior to the methods.

**This has been fixed.**

2) Introduction: The newly added paragraph about antibiotics (lines 89-97) rather reads like a Discussion. Consider to rephrase it and please move the sentence from line 94-97 to the Discussion section.

**We have moved line 94-97 to the Discussion section. We find that the part of this paragraph that remains serves as some background/introduction for the use of antibiotics for our study and would prefer to leave that information in the Introduction.**

3) For analysis of microglial cells and astrocytes (Methods section): Were there already other papers doing the same thing. If yes, references could be add.

**Yes. Thank you for pointing this out. We have now included a few different references in these sections.**

4) Figure 2: Would it be possible to add markers of statistically significant differences? This would make it easier for the reader.

**We now include indication of statistically significant differences for Figure 2.**

5) Figure 5: Please write the names with a capital letter first (Lactococcus etc.). Moreover, in lines 317-320 some mentioned genera are not found in the Figure (such as Lactobacillus). Please clarify.

**We have fixed the legend to include a capital letter for the genera. And, we have removed some of the sentences in the paragraph before Figure 5 in order to improve clarity. Originally, we were referring to the genera of bacteria that did not reveal a statistically significant difference between antibiotic-treated and normal drinking water. However, the graph, as you point out, does not clearly show this due to the very high relative abundance of *Lactococcus* for all antibiotic-treated groups. Therefore, we will keep it to a simple statement and let the Figure as well as the table that follows reveal the rest of the information.**

6) Legend Figure 6: There is no legend for F.

**Thank you for catching this error. This has been fixed.**

7) Sentence line 401: “Overall, Male and Female LF Antibiotics revealed decreased GFAP density”….this is a bit misleading and therefore “…reaching statistical significance in the female LF group.” should be added.

**We have revised this sentence and now include this clarification.**

8) The sentence in line 403-404 can be removed.

**We have removed this sentence.**

9) The Discussion should start with a short paragraph describing the main findings of the study.

**We have a short paragraph describing the main findings of the study in the Conclusion section that was added. In order to avoid being redundant, we would prefer to keep the Discussion and Conclusion section as they are.**
